# Perceived risk of substance use and associations with early experimentation: A latent profile analysis using ABCD study data

**DOI:** 10.1016/j.dadr.2026.100429

**Published:** 2026-03-14

**Authors:** Alejandra Fernandez, Deanna M. Barch, Micah E. Johnson, Hugh Garavan, Alexandra S. Potter, Sarahjane L. Dube, Nicholas Allgaier, Ethan T. Hunt, Miguel Ángel Cano, Sandra Estrada Gonzalez

**Affiliations:** a University of Texas Southwestern Medical Center, O’Donnell School of Public Health, Dallas, TX, United States; b Washington University in St. Louis, Departments of Psychological & Brain Sciences, Psychiatry, and Radiology, St. Louis, MO, United States; c University of California Los Angeles, Department of Family Medicine, Los Angeles, CA, United States; d University of Vermont, Department of Psychiatry, Burlington, VT, United States; e University of Texas Health Science Center at Houston (UTHealth) School of Public Health, Department of Health Promotion and Behavior Science, Austin, TX, United States; f The University of Texas Rio Grande Valley (UTRGV), Edinburg, TX, United States

**Keywords:** Latent profile analysis, Perceived harm, Substance use, Emerging adolescent, Experimentation

## Abstract

**Objective:**

The current study aims to determine heterogenous latent profiles of substance use perceived harm and examine the concurrent associations between profiles and substance use experimentation.

**Methods:**

We used data from the Adolescent Brain Cognitive Development^SM^ Study (5.1 data release) 1-year follow up. Participants (N = 11184) were 52.2% male between the ages of 9 and 14 (*M*_age_ = 10.48, *SD* = 0.65). Latent profile analysis was used with 11 perceived harm indicators. Multinomial logistic regression was used to determine the association between profile membership and substance use experimentation (i.e., alcohol sipping, tobacco puffing, and cannabis puffing).

**Results:**

A four-profile solution fit the data best. The four profiles included participants in 1) high harm perceptions across all substances, 2) low harm perceptions across all substances, 3) moderate harm perceptions across all substances, and 4) selective high-harm perceptions for some substances. Those in the low harm profile (AOR = 4.99, p < .01) and selective high-harm profile (AOR = 3.04, p < .05) were more likely to report puffing tobacco compared to the high harm profile. Also, those in the moderate harm profile (AOR = 1.36, p < .05) and those in the selective high-harm profile (AOR = 1.56, p < .001) were more likely to report sipping alcohol compared to the high harm group.

**Conclusion:**

The four-profile solution illustrates meaningful heterogeneity in how youth perceive substance-related harm, suggesting the need for more tailored prevention approaches rather than one-size-fits-all messaging considering that even partial recognition of risk may not fully protect against experimenting with substances.

## Highlights

• Very few studies examine perceived harm and substance use experimentation (e.g., sipping alcohol, puffing tobacco/marijuana) in ages 9-10.
• Four heterogenous profiles emerged using 11 perceived risk indicators across substances and varying levels of use intensity.
• Adolescents perceiving low harm, or low harm for light use but high harm for heavy use, were more likely to report experimentation.

## 1 Introduction

### 1.1 Substance use public health concern

Adolescent substance use behaviors are a public health concern in the United States (U.S.) associated with adverse physical, behavioral, social, and health-related outcomes, including visits to emergency rooms (White et al., 2018), substance use disorders (Jordan and Andersen, 2017), and suicidal thoughts and behaviors (Tervo-Clemmens et al., 2024). By the time an adolescent enters high school, approximately 15% report that they currently drink alcohol, 9% report current cannabis use, and 18% report they currently use an electronic vapor product (Hoots, 2023, Oliver, 2023). Further, prior to entering high school, some adolescents may engage in experimentation of substance use or low-level use (Sargent et al., 2017, Sullivan et al., 2022, Watts et al., 2021). For instance, in a U.S. national cohort of children and adolescents (*M*_age_ = 9.47 at baseline), findings indicated that at baseline, 22.5% had sipped alcohol, 0.68% had puffed nicotine, though only < 0.1% had puffed or tasted cannabis (Sullivan et al., 2022). Experimentation during late childhood and early adolescence (9–10 years of age) may be an important indicator of later adolescent substance using behaviors. For example, alcohol sipping is associated with subsequent alcohol and substance using behaviors, such as increased drinking frequency, greater consumption of alcohol in a single sitting, and alcohol-related social/behavioral concerns (Colder et al., 2018, Jackson et al., 2015). Thus, it is imperative to examine risk factors associated with late childhood/early adolescent substance use experimentation.

### 1.2 Theoretical framework

One risk factor associated with substance use and experimentation is the perception of harm associated with the behavior. As noted in the literature, the perception of harm due to engaging in a certain behavior is important to consider in either preventing or changing that behavior (Ferrer and Klein, 2015). Further, individuals can form their own risk perceptions, which in turn, have further health implications (Ferrer and Klein, 2015). For example, if an individual perceives they have low risk for a certain health outcome and they are, in fact, at low risk, behavioral change is not necessary. However, if an individual perceives themselves to be at low risk for a certain health outcome, yet they are at high risk, this can deter an individual from engaging in protective behaviors that may mitigate risk (Gaube et al., 2019). For example, in a study with 267 adolescents (12–20 years), those that reported vaping also reported a 35% lower perceived threat compared to those that did not vape (Gilmore et al., 2023). Further, among those that vaped regularly, some also reported regular cigarette use. Vaping is associated with cigarette use and nicotine addiction (Vogel et al., 2019), thus, perceived low risk of vaping may deter adolescents from engaging in protective behaviors against the use of other tobacco and nicotine products. In the case of perceived harm from substances, low perceived harm may encourage engagement in substance using behaviors. Their perceptions of low risk may place them at risk for subsequent substance use or substance use disorders.

### 1.3 Perceived harm

Perceived harm of substance use has been negatively associated with later substance use behaviors (Cleveland et al., 2008, Wright et al., 2016, Zimmerman and Farrell, 2017). For example, for alcohol use, adolescents who do not perceive risk when engaging in drinking are more likely to drink (Maslowsky et al., 2019), with most adolescents who abstained from drinking behaviors perceiving high doses of daily alcohol consumption as risky (Chomynova et al., 2009). For other substances, such as nicotine (e.g., use of e-cigarettes, vaping) (Amrock et al., 2015, Donaldson et al., 2021, Margolis et al., 2021), tobacco (Roditis et al., 2016, Strong et al., 2019), and cannabis (Chadi et al., 2020), existing research suggests a similar association, such that lower perceived harm was associated with current or ever use or being susceptible to use . However, there are some gaps in the literature that this papers aims to fill, including that few studies that have focused on early adolescent populations (i.e., starting at age 10) (Carliner et al., 2017) with most of the studies cited here having older adolescent samples (e.g., high school aged youth). Further, few studies have examined the association between perceived harm of multiple substances (Lardier et al., 2024, Liu et al., 2024) at varying intensities (Connell et al., 2010, Cummins and Lu, 2022) and substance use experimentation, as most research focused on perceived harm of one substance and/or regular substance use (e.g., regular use of cigarettes) or heavier use (e.g., five or more alcohol drinks in a row).

### 1.4 Profiles of perceived harm

Existing research is also limited because it may not be able to capture the dimensionality of perceived harm across multiple substances. Latent profile analysis is considered a person-centered approach versus a variable-centered approach (which is most often found in the literature) and a modeling technique that identifies unobserved mutually exclusive subgroups of individuals within a population (Berlin et al., 2014) and allows investigation of the heterogeneity among the population of interest. Latent profile analysis can be used to study patterns and the multidimensionality of harm perceptions across multiple substances simultaneously, rather than examining the association between the harm perception of each substance and each substance use experimentation outcome. This is particularly relevant given the multicollinearity that may exist when examining multiple substances concurrently. Further, adolescents' perceived harm may vary based on type of substance with different types of substances being perceived as involving more or less risk than others. For this reason, a person-centered approach to examining patterns of perceived risk across multiple substances and intensity/frequency of use can add to the existing literature. Although existing research in this area has applied a latent profile approach, the literature is limited because it has been focused on adult populations (Tripathi et al., 2024), used perceived harm to predict profiles of substance using behaviors (Scheier and Komarc, 2020), or did not examine substance use experimentation as an outcome of memberships in profiles of perceived harm (Kong et al., 2019).

### 1.5 Current study

Given the gaps in the literature, including limited evidence found on the effects of perceived harm and substance use experimentation among early adolescents and the use of variable-centered approaches rather than a person-centered approach, the aims of the current study are to: 1) determine whether there are heterogenous latent profiles of perceived harm of substance-using behaviors and 2) examine the association between latent profile membership and concurrent substance use experimentation. We hypothesize that there will be more than two profiles identified via latent profile analysis, such that compared to either the presence or absence of harm, we expect more than two profiles due to the variability in perceived harm that may surface across multiple substances. We also hypothesize that higher perceived harm will be associated with lower likelihoods of substance use experimentation, with potentially different associations across latent profiles as a function of which substance youth perceive as harmful or having negative consequences.

## 2 Methods

### 2.1 Participants

Data from the Adolescent Brain Cognitive Development^SM^ Study (ABCD Study®; 5.1 data release) were analyzed. The ABCD Study is the largest longitudinal cohort study of U.S. youth. The study began in 2016–2018 (baseline, Year 0) and 11,878 9–10 years old from 21 sites across the U.S. were recruited. Additional details regarding ABCD Study recruitment have been previously published (Garavan et al., 2018). There is limited existing literature examining perceived risk and substance use experimentation specifically during emerging early adolescence. Prior studies have largely focused on middle to late adolescence; thus, we aim to address this gap by examining cross-sectional associations during this emerging developmental stage using the 1-year follow-up parent- and adolescent-reported data, except for biological sex which was assessed at baseline. The University of California, San Diego (UCSD) provided central institutional review board approval. Parents provided consent and adolescents provided assent.

### 2.2 Measures

*Substance use experimentation (Year 1 Follow Up).* To assess substance use experimentation (Jackson et al., 2015), adolescents completed the iSay Sipping Inventory. Participants were asked to respond to three items, “Have you ever tried at any time in your life? A sip of alcohol such as beer, wine or liquor (rum, vodka, gin, whiskey)”, “Have you ever tried at any time in your life? A puff from a tobacco or electronic cigarette, or vape pens, or e-hookah”, and “Have you ever tried at any time in your life? A puff or eaten any marijuana, also called pot, grass, weed or ganja?” Response options included, 0 =  No, 1 =  Yes. Note, although response options use the term marijuana, the term cannabis will be used hereafter.

*Latent profile indicators: Perceived Harm of Substance Use (Year 1 Follow Up)*. Adolescent participants were asked to respond to 11 items that were used to assess perceived harm of substance-using behaviors (Johnston et al., 2017). Participants were asked to respond to items such as, “How much do you think people risk harming themselves (physically or in other ways) if they …” with behaviors including consuming alcohol beverages, cigarette use, e-cigarette use, smokeless tobacco, and marijuana use with varied levels of severity. Response options ranged from 0 =  No Risk to 3 =  Great Risk. See Supplementary Table for list of indicators. Note, although response options use the term marijuana, the term cannabis will be used hereafter.

*Covariates/control variables*. Sex assigned at birth (baseline: male and female), parent-reported youth race/ethnicity (White, Black, Hispanic, Asian, Another Race or Ethnicity; 1-Year Follow Up), parent-reported combined family income (≤ $49,999, $50,000 to $199,999, ≥ $200,000; 1-Year Follow Up),and adolescent age (in years; 1-Year Follow Up) were chosen due to their association with substance use behaviors (Probst et al., 2014, SAMHSA, 2024, White, 2020).

### 2.3 Data analysis

To address Aim 1, latent profile analysis (LPA) was used to identify heterogeneous profiles of adolescents based on their responses to the 11 perceived harm indicators. We estimated 2, 3, and 4 profile models using the R packages tidyLPA, mice, purr, tidyr, and openxlsx (Rosenberg et al., 2019) with equal variance and zero covariance (Model 1; the most constrained and restrictive model). Model estimation and fit were compared using Akiake information criterion (AIC; the model with lower values is preferred), Bayesian information criterion (BIC; the model with lower values is preferred), entropy (values approaching 1.0 indicate better fit), and interpretability of profile membership to identify the model with the best fit to the data (Weller et al., 2020).

Once the best estimated/preferred model was selected, an omnibus chi-square test (for categorical) and F-test (for continuous) were used to compare all profiles on demographic and outcome variables, and when significant, follow up by pairwise test to determine which profile differed from the others.

To address Aim 2, participants' profile membership was used in a multinomial logistic regression analysis (SAS Proc SurveyLogistic) to assess the association between participants’ latent profile membership and the outcomes of interest, alcohol sipping, tobacco puffs, and cannabis puffs. Adjusted models (including covariates and confounding variables) were examined. Results were considered significant at the *p* < .05 level.

Latent profile analysis was conducted in RStudio version 4.4.3 and logistic regression were performed in SAS version 9.4. Missing data across the 11 perceived harm indicators ranged from 6.10% to 26.99%. Missing data across the substance use experimentation items ranged from 1.58% to 21.67%. Missing data was accounted for by using multiple imputation prior to latent profile analysis. ACS ranked propensity scores were used for weighting and Site ID was used as a strata.

## 3 Results

### 3.1 Descriptives

Descriptive statistics, including variable frequencies, means, and standard deviations are provided in Table 1. Demographic characteristics of the study sample include a mean age of 10.48 years old (*SD* =.65), approximately half (52.2%) of the sample reporting their biological sex at birth as male, a majority reporting their race/ethnicity as White (53.4%), and a combined family income of $50,000 to $199,999 for the majority of the sample (59.7%). Reported substance use experimentation was 9.7% (n = 1083) for sipping alcohol such as beer, wine, and liquor, 0.4% (n = 45) for puff of tobacco, e-cig, juul, vape pen, e-hookah, cigar, or pipe, and 0.1% (n = 12) for puff or eaten cannabis. Additional details are found in Table 1.

**Table 1:** Demographics and descriptive statistics at Year 1 follow up (n = 11184).

	Mean	SD	N	%	Missing
Sex					0 (0.0%)
Male			6191	52.2%	
Female			5677	47.8%	
Adolescent Age (years)	10.48	0.65			26 (0.2%)
Race/Ethnicity					7 (0.1%)
White			5963	53.4%	
Black			1584	14.2%	
Hispanic			2217	19.8%	
Asian			241	2.2%	
Another race/ethnicity			1172	10.5%	
Combined Family Income					844 (7.5%)
≤ $49,999			2809	27.2%	
$50,000 to $199,999			6175	59.7%	
≥ $200,000			1356	13.1%	
Religious Sipping					10103 (90.3%)
No			400	37.0%	
Yes			681	63.0%	
Perceived Harm					
…if they try one or two drinks of an alcohol beverage (beer, wine, liquor)?	1.66	.95			1111 (9.9%)
…if they take one or two drinks nearly every day?	2.22	.87			828 (7.4%)
…if they have five or more drinks of an alcohol beverage, once or twice each weekend?	2.51	.78			767 (6.8%)
…if they try one or two cigarettes?	1.87	.95			850 (7.6%)
…if they smoke cigarettes occasionally?	2.38	.84			747 (6.6%)
…if they are smoking one or more packs of cigarettes per day?	2.79	.64			682 (6.0%)
…if they use e-cigarettes regularly?	2.45	.83			1879 (16.8%)
…if they use smokeless tobacco regularly?	2.48	.81			1759 (15.7%)
…if they try cannabis* once or twice?	2.13	.92			3019 (26.9%)
…if they use cannabis* occasionally?	2.51	.79			2936 (26.2%)
…if they use cannabis* regularly?	2.72	.69			2910 (26.0%)
Sip of alcohol such as beer, wine, or liquor					177 (1.5%)
No			9924	90.2%	
Yes			1083	9.7%	
Puff of tobacco, e-cig, juul, vape pen, e-hookah, cigar, or pipe					287 (2.5%)
No			10852	99.6%	
Yes			45	0.4%	
Puff or eaten any cannabis*					2424 (21.6%)
No			8748	99.9%	
Yes			12	0.1%	

Note: Variable from Year 0: sex at birth. Perceived Harm Scores ranged from 0 (No Risk) to 3 (Great Risk); * indicates that although the term “cannabis” is used in this table, the original measure item used the term “marijuana”

### 3.2 Latent profile analysis

**Fig. 1:**
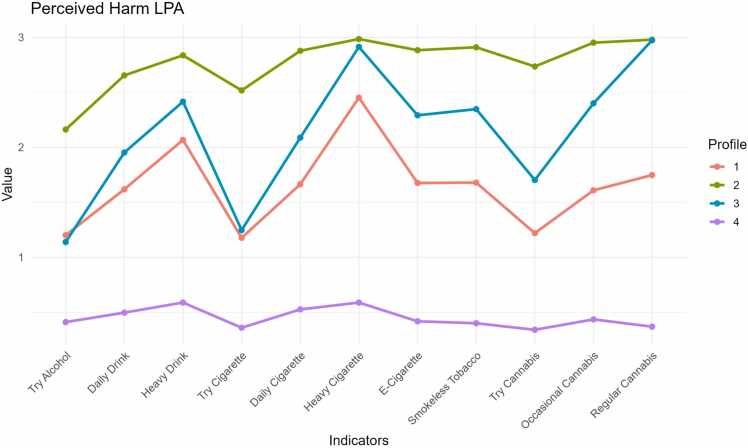
Latent profile 4 groups. Note: Class designations: 1: Moderate harm perceptions across all substances; 2: High harm perceptions across all substances; 3: Selective high-harm perceptions; 4: Low harm perceptions across all substances; Values ranged from 0 =  No Risk, 1 =  Slight Risk, 2 =  Moderate Risk, 3 =  Great Risk.

After assessing fit according to fit indices (Table 2), the four-profile solution (AIC = 208159.61, BIC = 208584.30, entropy =.93) was chosen as the best fitting model with the lowest values for the AIC and BIC fit indices and interpretability of the 4-profile solution compared to the 2- and 3-profile solution. The four-profile solution (see Fig. 1) was composed of the following profiles: 1) high harm perceptions across all substances (adolescents who generally perceived moderate to great risk for all substances and intensity of substance use; *n* = 5969), 2) low harm perceptions across all substances (adolescents who generally perceived no risk to slight risk for all substances and intensity of substance use; *n* = 589), 3) moderate harm perceptions across all substances (adolescents who perceived a slight or moderate amount of risk for most substances and intensity of substance use; *n* = 1250), and 4) selective high-harm perception (adolescents who perceived slight-low risk for low level use of alcohol, cigarettes, and cannabis with moderate-great risk for heavy alcohol, regular and heavy cigarette use, occasional and regular cannabis use and regular e-cigarette and smokeless tobacco use; *n* = 3376). Specifically, those in the selective high-harm profile perceived moderate to great risk for heavy use, such as “five or more drinks of an alcohol beverage once or twice each weekend”, “smoking one or more packs of cigarettes per day”, and “using cannabis regularly”, “use of e-cigarettes”, and “use of smokeless tobacco.” Alternatively, these same adolescents, perceived only slight risk for lower-level use, such as “if trying one or two drinks ever”, “trying one or two cigarettes”, or “trying cannabis once or twice.” There were significant differences in demographics, specifically adolescent age, race/ethnicity, combined family income, religious sipping, and substance use experimentation across profiles (see Table 3).

**Table 2:** LPA fit indices.

Fit Statistic	2 Profiles	3 Profiles	4 Profiles
AIC	248508.7612	219462.137	208159.619
BIC	248757.717	219798.96	208584.3086
Entropy	0.99527667	0.94102939	0.9313173
Group size (n, %)			
Class 1	10444	7326	5969
Class 2	740	3267	3376
Class 3		591	1250
Class 4			589
Class 5

**Table 3:** Group differences across four profiles.

	Low Harm (n = 589)	Moderate Harm(n = 1250)	High Harm(n = 5969)	Selective High-Harm(n = 3376)	Chi-square/F-test
Sex					χ^2^ = 2.173, p =.53
Male	315	634	3113	1787	
Female	274	616	2856	1589	
Adolescent Age (years)	10.31^abc^	10.46^c^	10.48^a^	10.51^b^	F-value = 15.77, p <.001
Race/Ethnicity					χ^2^ = 338.07, p <.001
White	170^def^	620^d^	3142^e^	2031^cd^	
Black	189^abce^	241^abcd^	800^bd^	354^ac^	
Hispanic	147^cf^	237^c^	1276^cde^	557^bd^	
Asian	8^b^	18^b^	149^ab^	66	
Another race/ethnicity	74^ad^	133^a^	599^ac^	366^ab^	
Combined Family Income					χ^2^ = 255.02, p <.001
≤ $49,999	279^ac^	368^ac^	1472	690^ac^	
$50,000 to $199,999	220^ab^	642^a^	3347	1966^ab^	
	28^bc^	127^bc^	700	501^bc^	
Religious Sipping					χ^2^ = 16.996, p <.001
Yes	30	104	255^a^	292^a^	
Sip of alcohol such as beer, wine, or liquor					χ^2^ = 72.513, p <.001
Yes	45^cd^	153^bd^	457^ab^	428^ac^	
Puff of tobacco, e-cig, juul, vape pen, e-hookah, cigar, or pipe					χ^2^ = 16.701, p <.001
Yes	7^a^	7	13^a^	18	
Puff or eaten any cannabis*					χ^2^ = 18.776, p <.001
Yes	3^a^	4	2^a^	3	

Note: Some demographic variable totals do not add up to Class variable totals (rows) due to missingness at the demographic variable level. Values with the same superscripts letters differ significantly based on pairwise chi-square test and F-test p-values (p <.05)

* indicates that although the term “cannabis” is used in this table, the original measure item used the term “marijuana”

### 3.3 Concurrent associations with substance use experimentation

We examined the association between profile membership and concurrent substance use experimentation (i.e., sipping alcohol, puffing tobacco, and puffing cannabis; see Table 4) in models adjusted for covariates/confounding variables. Age was associated with an increased likelihood of experimentation (sipping: AOR = 1.25, Confidence Interval [CI] = 1.10, 1.42, *p* < .001, puffing tobacco: AOR = 2.58, CI = 1.39, 4.77, p < .01, puffing cannabis: AOR = 4.64, CI = 1.64, 13.16, p < .01). Sex assigned at birth was associated with experimentation, with females reporting lower odds of sipping alcohol (AOR =.82, CI =.70,.97, p < .05). Compared to White participants, Black (AOR =.37, CI =.26,.52, p < .001), Hispanic (AOR =.50, CI =.38,.66, p < .001), and those reporting Another Race/Ethnicity (AOR =.67, CI =.49,.91, p < .05) were less likely to report sipping alcohol. Compared to those who made more than $200,000, those who made less than $49,999 (AOR =.47, CI =.35,.63, p < .001) and those reporting an income between $50,000 and $199,999 (AOR =.69, CI =.57,.85, p < .001) were less likely to report sipping alcohol. Findings indicated that, compared to those in the high harm profile, those in the low harm profile were more likely to report puffing tobacco (AOR = 4.99, CI = 1.46, 17.01, *p* < .01). Additionally, compared to those in the high harm profile, those in the moderate harm profile, were more likely to report sipping alcohol (AOR = 1.36, CI = 1.04, 1.75, *p* < .05). Finally, compared to those in the high harm profile, those in the selective high-harm profile, were more likely to report sipping alcohol (AOR = 1.56, CI = 1.30, 1.87, *p* < .001) and puffing tobacco (AOR = 3.04, CI = 1.27, 7.26, *p* < .05).

**Table 4:** Experimentation by class: Adjusted odds ratios.

	High Harm	Low Harm				Moderate Harm				Selective High-Harm			
		Estimate	AOR	CI	p-value	Estimate	AOR	CI	p-value	Estimate	AOR	CI	p-value
Sipping Alcohol	Ref	.3096	1.363	.897–2.070	NS	**.3037**	**1.355**	**1.047–1.754**	**.0211**	**.4469**	**1.564**	**1.305–1.873**	**< .0001**
Puff Tobacco	Ref	**1.6091**	**4.998**	**1.468–17.015**	**< .01**	.6887	1.991	.580–6.831	NS	**1.1118**	**3.040**	**1.272–7.267**	**< .05**
Puff Cannabis*	Ref	2.1108	8.255	.971–70.174	NS	1.7281	5.630	.817–38.786	NS	1.3921	4.023	.626–25.861	NS

Note: Bold values indicate significance at p < .05; * indicates that although the term “cannabis” is used in this table, the original measure item used the term “marijuana”

## 4 Discussion

As previously stated, perceived harm is shown to be associated with health behaviors, however, less research has examined perceived harm of substance use among early adolescents and its association with substance use experimentation. This study aimed to apply a latent profile analysis method to examine distinct profiles of perceived harm across multiple substances at varying levels of use, while also examining how membership across different profiles may be associated with concurrent substance use experimentation. This study demonstrates that there is heterogeneity in adolescent perceived harm across multiple substances which, in turn, is associated with substance use experimentation. This study adds to the existing literature by suggesting that perceived harm may differ not just across substance but also across intensity of use, which may play a role in adolescents’ experimentation behaviors. It may be important to consider the observed heterogeneity of perceived harm across substances when developing prevention and intervention programming among early adolescents.

Findings show that four unique profiles emerged, low harm, moderate harm, high harm, and selective high-harm risk perceptions. The high harm profile was the largest profile in this sample, which may be expected considering the age range of the adolescents, i.e., 8–11 years of age. At this age, adolescents are more likely to hear from adults about the negative consequences of substance use (Kelly et al., 2002) and may be less exposed to peers who engage in substance use (Cambron et al., 2018) who may influence their harm perceptions of substance use. Alternatively, the smallest profile (*n* = 589), of low harm perceptions, suggests that a small portion of our sample perceived no harm with any of the substances and at any level of intensity. In our study, adolescents in this profile were slightly younger than those in the high harm profile, contrary to what has been found in other studies (Liu et al., 2024). A reason for this inconsistency may be due to lack of knowledge related to substance use harm. As previously stated, adolescents primarily get information related to substance use from parents and, it is possible for some youth, that parent-adolescent conversations regarding substance use may not take place during childhood or early adolescence due to parents believing that adolescents are not yet engaging in substance use this early in life (Guilamo-Ramos et al., 2006; Parents Say, 2011), which may be a misconception. For example, according to the Centers for Disease Control and Prevention ((CDC), 1991–2023), approximately 13% of surveyed adolescents reported having their first drink of alcohol before the age of 13. Family-based interventions may help in informing parents that some adolescents begin engaging in substance use behaviors before entering high school.

A unique contribution of this study is the identification of the profile selective high-harm profile. This group was distinct from the others, particularly the moderate harm group, because adolescents in the selective high-harm group perceived low harm at low levels of substance use but had similar harm perceptions to those in the high harm profile for heavier use substance using behaviors, such as “five or more drinks”, “smoking one or more packs a day”, or “use cannabis regularly.” Belonging to this profile may suggest that adolescents believe that substance use experimentation (or low-level use) is not dangerous. Previous studies have shown that when adolescents sip alcohol, it is often supplied by a parent (Aiken et al., 2020). For example, one study showed that 14.6% of adolescents reported that sips were supplied by a parent, while only 6.9% reported sips were supplied by others (Aiken et al., 2020). To adolescents, parental supply may suggest a certain level of safety in taking a sip from a parent’s drink. However, adolescents may also be receiving messaging from adults about the negative consequences (e.g., asthma and inability to engage in physical activity) of heavier substance using behaviors (Small et al., 2012), which may shape their perceptions that heavy substance use can lead to negative outcomes. It is noteworthy that adolescents in this profile did not perceive use of e-cigarettes as having a great risk (i.e., instead it was perceived as moderately risky). E-cigarette products have been considered a harm reduction strategy (Feeney et al., 2022) in combating the use of combustible tobacco products (e.g., cigarettes), which may influence adolescents to perceive these products as less harmful or addictive.

Aligned with previous research, our findings suggest that perceived harm was associated with concurrent substance use experimentation. As expected, findings indicated that adolescents in the low harm perceptions were more likely to engage in puffing tobacco compared to adolescents who perceived high harm for substance use at any level of use. Although adolescents may receive messaging relating to the harms of substance use, some adolescents may not be exposed to the same messaging or knowledge related to the adverse effects of substance use (Pettigrew et al., 2018), perhaps leading to lower perceived harm and experimentation.

Additionally, adolescents who perceived low harm for substance use experimentation (i.e., trying a substance) but perceived high harm for heavier substance using behaviors (i.e., regular use; i.e., selective high-harm group), were more likely to report sipping alcohol and puffing tobacco (1.5x more likely and 3x more likely, respectively). A unique finding of this study may be regarding the association with engaging in puffing tobacco, e-cigarettes, Juul, vape pen, e-hookah, cigar, or pipe. Although this profile was composed of adolescents who perceived heavy tobacco use (i.e., smoking more than one pack a day) as highly harmful, they perceived use of e-cigarettes and smokeless tobacco as slightly less harmful (although still a moderate risk). The perception of less harm for e-cigarettes (and related electronic vaping methods) may be associated with their increased puffing if considered less harmful. Due to multiple methods of consumption being grouped within the puffing variable, this may hinder the ability to distinguish between adolescents engaged in puffing tobacco versus e-cigarettes. Nevertheless, perceptions of less harm for substances such as e-cigarettes and smokeless tobacco may be due to misperceptions related to e-cigarettes. For example, beliefs that e-cigarettes and/or Juul products do not contain nicotine (Initiative, 2018). These misperceptions of e-cigarettes and other smokeless versions of tobacco may make these products seem less harmful, leading to experimentation, which may in turn lead to heavier substance use because of their addictive properties.

### 4.1 Limitations

Study strengths include use of a nationally diverse sample of early adolescents, the application of a latent profile approach to examine the multidimensionality of perceived risk and substance using behaviors, and the use of multiple imputation to account for missing data. However, study limitations may affect the interpretability of the results. First, although the ABCD Study dataset is composed of multiple waves of data, this study examined perceived harm perceptions at the year 1 follow up (first time adolescents were asked these questions) and concurrent substance use experimentation, which makes the analysis cross-sectional. This limits the ability to make causal interpretations. Second, the low prevalence of substance use experimentation at the year 1 follow up, particularly among the puffing tobacco and puffing cannabis outcomes, may inflate the odds ratios. Future studies may examine the association between perceived harm profiles and substance using behaviors as adolescents become older and engage in heavier alcohol, tobacco, nicotine, and cannabis use.

## 5 Conclusions

The findings from this study indicate that distinct profiles of perceived harm are associated with adolescent substance use experimentation, particularly sipping alcohol and smoking tobacco. Although results do not suggest a causal relationship, they offer important considerations for prevention and intervention efforts. Specifically, interventions may benefit from integrating messaging not only related to the harms of heavy substance use (East et al., 2026, Esrick et al., 2019) but those surrounding low-dose or occasional experimentation (i.e., subsequent associations with heavier use). Intervention messaging that focuses exclusively on the dangers of heavy use may be insufficient for adolescents who differentiate between experimentation and heavier use and perceive the former as relatively low risk. Tailoring intervention content to address this distinction may help reduce early substance use behaviors among adolescents who otherwise endorse high perceived harm.

## CRediT authorship contribution statement

**Miguel Ángel Cano:** Writing – review & editing. **Sandra Estrada Gonzalez:** Writing – review & editing. **Ethan T. Hunt:** Writing – review & editing. **Deanna M. Barch:** Writing – review & editing, Investigation. **Micah E. Johnson:** Writing – review & editing, Funding acquisition. **Alejandra Fernandez:** Writing – review & editing, Writing – original draft, Visualization, Formal analysis, Conceptualization. **Sarahjane** **L. Dube:** Writing – review & editing, Investigation, Data curation. **Nicholas Allgaier:** Writing – review & editing, Investigation. **Hugh Garavan:** Writing – review & editing, Investigation, Funding acquisition. **Alexandra S. Potter:** Writing – review & editing, Investigation, Funding acquisition.

## Funding

10.13039/100000026NIDA R25DA059073 (PIs: Johnson, Garavan, Potter); NHLBI K01HL166439–01A1 (PI: Fernandez); NCATS UL1 TR003163 (PI: Toto).

## Declaration of Competing Interest

The authors declare the following financial interests/personal relationships which may be considered as potential competing interests: Alejandra Fernandez reports financial support was provided by National Heart Lung and Blood Institute, and statistical analysis and travel were provided by National Institute on Drug Abuse. All the other authors declare that they have no known competing financial interests or personal relationships that could have appeared to influence the work reported in this paper.

## References

[bib1] (CDC), C. f. D. C. a. P. (1991-2023). *1991-2023**High School Youth Risk Behavior Survey Data* 〈https://yrbs-explorer.services.cdc.gov/#/graphs?questionCode=H41&topicCode=C03&location=XX&year=2023〉

[bib2] Aiken A., Clare P.J., Boland V.C., Degenhardt L., Yuen W.S., Hutchinson D., Najman J., McCambridge J., Slade T., McBride N., De Torres C., Wadolowski M., Bruno R., Kypri K., Mattick R.P., Peacock A. (2020). Parental supply of sips and whole drinks of alcohol to adolescents and associations with binge drinking and alcohol-related harms: a prospective cohort study. Drug Alcohol Depend..

[bib3] Amrock S.M., Zakhar J., Zhou S., Weitzman M. (2015). Perception of e-cigarette harm and its correlation with use among US adolescents. Nicotine & Tob. Res..

[bib4] Berlin K.S., Williams N.A., Parra G.R. (2014). An introduction to latent variable mixture modeling (part 1): overview and cross-sectional latent class and latent profile analyses. J. Pedia Psychol..

[bib5] Cambron C., Kosterman R., Catalano R.F., Guttmannova K., Hawkins J.D. (2018). Neighborhood, family, and peer factors associated with early adolescent smoking and alcohol use. J. Youth Adolesc..

[bib6] Carliner H., Brown Q.L., Sarvet A.L., Hasin D.S. (2017). Cannabis use, attitudes, and legal status in the US: A review. Prev. Med..

[bib7] Chadi N., Levy S., Weitzman E.R. (2020). Moving beyond perceived riskiness: Marijuana-related beliefs and marijuana use in adolescents. Subst. Abus..

[bib8] Chomynova P., Miller P., Beck F. (2009). Perceived risks of alcohol and illicit drugs: Relation to prevalence of use on individual and country level. J. Subst. Use.

[bib9] Cleveland M.J., Feinberg M.E., Bontempo D.E., Greenberg M.T. (2008). The role of risk and protective factors in substance use across adolescence. J. Adolesc. Health.

[bib10] Colder C.R., Shyhalla K., Frndak S.E. (2018). Early alcohol use with parental permission: Psychosocial characteristics and drinking in late adolescence. Addict. Behav..

[bib11] Connell C.M., Gilreath T.D., Aklin W.M., Brex R.A. (2010). Social-ecological influences on patterns of substance use among non-metropolitan high school students. Am. J. Community Psychol..

[bib12] Cummins K., Lu Y. (2022). Adolescents’ perceptions of substance use harms are contingent on mode of administration and type of substance. Subst. Abus. Res. Treat..

[bib13] Donaldson C.D., Fecho C.L., Ta T., Vuong T.D., Zhang X., Williams R.J., Roeseler A.G., Zhu S.H. (2021). Vaping identity in adolescent e-cigarette users: a comparison of norms, attitudes, and behaviors. Drug Alcohol Depend..

[bib14] East K., Simonavičius E., Taylor E.V., Brose L., Robson D., McNeill A. (2026). Interventions to change vaping harm perceptions and associations between harm perceptions and vaping and smoking behaviours: A systematic review. Addiction.

[bib15] Esrick J., Kagan R.G., Carnevale J.T., Valenti M., Rots G., Dash K. (2019). Can scare tactics and fear-based messages help deter substance misuse: a systematic review of recent (2005–2017). research. Drugs Education Prevention Policy.

[bib16] Feeney S., Rossetti V., Terrien J. (2022). E-Cigarettes-a review of the evidence-harm versus harm reduction. Tob. Use Insights.

[bib17] Ferrer R., Klein W.M. (2015). Risk perceptions and health behavior. Curr. Opin. Psychol..

[bib18] Garavan H., Bartsch H., Conway K., Decastro A., Goldstein R.Z., Heeringa S., Jernigan T., Potter A., Thompson W., Zahs D. (2018). Recruiting the ABCD sample: Design considerations and procedures. Dev. Cogn. Neurosci..

[bib19] Gaube S., Lermer E., Fischer P., Raue M., Streicher B., Lermer E. (2019). Perceived Safety: A Multidisciplinary Perspective.

[bib20] Gilmore B.A., Gilmore C.M., Reveles K.R., Koeller J.M., Spoor J.H., Flores B.E., Frei C.R. (2023). A Survey of Vaping Use, Perceptions, and Access in Adolescents from South-Central Texas Schools. Int. J. Environ. Res. Public Health.

[bib21] Guilamo-Ramos V., Jaccard J., Turrisi R., Johansson M., Bouris A. (2006). Maternal perceptions of alcohol use by adolescents who drink alcohol. J. Stud. Alcohol.

[bib22] Hoots B.E. (2023). Alcohol and other substance use before and during the COVID-19 pandemic among high school students—Youth Risk Behavior Survey, United States, 2021. MMWR Suppl..

[bib23] Initiative T. (2018). JUUL eCigar. gain Pop. Youth but Aware. nicotine Presence Remains Low. In..

[bib24] Jackson K.M., Barnett N.P., Colby S.M., Rogers M.L. (2015). The prospective association between sipping alcohol by the sixth grade and later substance use. J. Stud. Alcohol Drugs.

[bib25] Johnston L.D., O'Malley P.M., Miech R.A., Bachman J.G., Schulenberg J.E. (2017). Monit. Future Natl. Surv. Results Drug Use 19752016 Overv. key Find. Adolesc. Drug Use.

[bib26] Jordan C.J., Andersen S.L. (2017). Sensitive periods of substance abuse: Early risk for the transition to dependence. Dev. Cogn. Neurosci..

[bib27] Kelly K.J., Comello M.L., Hunn L.C. (2002). Parent-child communication, perceived sanctions against drug use, and youth drug involvement. Adolescence.

[bib28] Kong G., Simon P., Mayer M.E., Barrington-Trimis J.L., Pacek L.R., Cooper M., Guy M.C., Stanton C.A. (2019). Harm perceptions of alternative tobacco products among US Adolescents. Tob. Regul. Sci..

[bib29] Lardier D.T., Davis A.N., Verdezoto C.S., Cruz L., Magliulo S., Herrera A., Garcia-Reid P., Reid R.J. (2024). Latent class groups of concurrent substance use among adolescents in an urban community: correlates with mental health, access to drugs and alcohol, and risk perception. Subst. Use Addctn J..

[bib30] Liu J., McCauley D., Gaiha S.M., Halpern-Felsher B. (2024). Perceptions of harm and addictiveness for nicotine products, thc e-cigarettes, and e-cigarettes with other ingredients among adolescents, young adults, and adults. Subst. Use Misuse.

[bib31] Margolis K.A., Thakur S.K., Nguyen Zarndt A., Kemp C.B., Glover-Kudon R. (2021). E-cigarette susceptibility among u.s. middle and high school students: national youth tobacco survey data trend analysis, 2014-2018. Prev. Med.

[bib32] Maslowsky J., Owotomo O., Huntley E.D., Keating D. (2019). Adolescent risk behavior: differentiating reasoned and reactive risk-taking. J. Youth Adolesc..

[bib33] Oliver B.E. (2023). Electronic vapor product use among high school students—youth risk behavior survey, United States, 2021. MMWR Suppl..

[bib34] Parents Say (2011). Other Teens Drink Use Marijuana But My Kids Don. ’t.

[bib35] Pettigrew J., Miller-Day M., Shin Y., Krieger J.L., Hecht M.L., Graham J.W. (2018). Parental messages about substance use in early adolescence: extending a model of drug-talk styles. Health Commun..

[bib36] Probst C., Roerecke M., Behrendt S., Rehm J. (2014). Socioeconomic differences in alcohol-attributable mortality compared with all-cause mortality: a systematic review and meta-analysis. Int J. Epidemiol..

[bib37] Roditis M., Delucchi K., Cash D., Halpern-Felsher B. (2016). Adolescents' perceptions of health risks, social risks, and benefits differ across tobacco products. J. Adolesc. Health.

[bib38] Rosenberg J.M., Beymer P.N., Anderson D.J., Van Lissa C., Schmidt J.A. (2019). tidyLPA: An R package to easily carry out latent profile analysis (LPA) using open-source or commercial software. J. Open Source Softw..

[bib39] SAMHSA (2024). Key Subst. Use Ment. Health Indic. U. S. Results 2023 Natl. Surv. Drug Use Health.

[bib40] Sargent J.D., Gabrielli J., Budney A., Soneji S., Wills T.A. (2017). Adolescent smoking experimentation as a predictor of daily cigarette smoking. Drug Alcohol Depend..

[bib41] Scheier L.M., Komarc M. (2020). Are E-cigarette users a unique group of smokers? latent class analysis of the National Youth Tobacco Survey. J. Drug Educ..

[bib42] Small S.P., Eastlick Kushner K., Neufeld A. (2012). Dealing with a Latent Danger: Parents Communicating with Their Children about Smoking. Nurs. Res Pr..

[bib43] Strong D.R., Messer K., White M., Shi Y., Noble M., Portnoy D.B., Persoskie A., Kaufman A.R., Choi K., Carusi C., Bansal-Travers M., Hyland A., Pierce J. (2019). Youth perception of harm and addictiveness of tobacco products: findings from the population assessment of tobacco and health study (Wave 1). Addict. Behav..

[bib44] Sullivan R.M., Wade N.E., Wallace A.L., Tapert S.F., Pelham W.E., Brown S.A., Cloak C.C., Feldstein Ewing S.W., Madden P.A.F., Martz M.E., Ross J.M., Kaiver C.M., Wirtz H.G., Heitzeg M.M., Lisdahl K.M. (2022). Substance use patterns in 9–13-year-olds: longitudinal findings from the adolescent brain cognitive development (ABCD) study. Drug Alcohol Depend. Rep..

[bib45] Tervo-Clemmens B., Gilman J.M., Evins A.E., Bentley K.H., Nock M.K., Smoller J.W., Schuster R.M. (2024). Substance use, suicidal thoughts, and psychiatric comorbidities among high school students. JAMA Pediatr..

[bib46] Tripathi O., Parada H., Shi Y., Matt G.E., Quintana P.J., Liles S., Bellettiere J. (2024). Perception of harm is strongly associated with complete ban on in-home cannabis smoking: a cross-sectional study. BMC Public Health.

[bib47] Vogel E.A., Prochaska J.J., Ramo D.E., Andres J., Rubinstein M.L. (2019). Adolescents' e-cigarette use: increases in frequency, dependence, and nicotine exposure over 12 Months. J. Adolesc. Health.

[bib48] Watts A.L., Wood P.K., Jackson K.M., Lisdahl K.M., Heitzeg M.M., Gonzalez R., Tapert S.F., Barch D.M., Sher K.J. (2021). Incipient alcohol use in childhood: Early alcohol sipping and its relations with psychopathology and personality. Dev. Psychopathol..

[bib49] Weller B.E., Bowen N.K., Faubert S.J. (2020). Latent class analysis: a guide to best practice. J. Black Psychol..

[bib50] White A.M. (2020). Gender Differences in the Epidemiology of Alcohol Use and Related Harms in the United States. Alcohol Res.

[bib51] White A.M., Slater M.E., Ng G., Hingson R., Breslow R. (2018). Trends in Alcohol-Related Emergency Department Visits in the United States: Results from the Nationwide Emergency Department Sample, 2006–2014. Alcohol. Clin. Exp. Res..

[bib52] Wright E.M., Fagan A.A., Pinchevsky G.M. (2016). Penny for your thoughts? The protective effect of youths’ attitudes against drug use in high-risk communities. Youth Violence Juv. Justice.

[bib53] Zimmerman G.M., Farrell C. (2017). Parents, peers, perceived risk of harm, and the neighborhood: contextualizing key influences on adolescent substance use. J. Youth Adolesc..

