# Supplementary material for: Perceived risk of substance use and associations with early experimentation: A latent profile analysis using ABCD study data
Source: Drug Alcohol Depend Rep. 2026 Mar 14;19:100429. doi: 10.1016/j.dadr.2026.100429 (PMC13022672; doi:10.1016/j.dadr.2026.100429)
Supplement: Supplementary file 1 — Supplementary material [file mmc1.docx]

Supplementary Table. Latent profile indicators

| **How much do you think people risk harming themselves (physically or in other ways) if they …** |
| --- |
| try one or two drinks of an alcoholic beverage (beer, wine, liquor)? |
| take one or two drinks nearly every day? |
| have five or more drinks of an alcoholic beverage, once or twice each weekend? |
| try one or two cigarettes? |
| smoke cigarettes occasionally? |
| are smoking one or more packs of cigarettes per day? |
| use e-cigarettes regularly? |
| use smokeless tobacco regularly? |
| try marijuana once or twice? |
| use marijuana occasionally? |
| use marijuana regularly? |

Note: Scores ranged from 0 = No Risk, 1 = Slight Risk, 2 = Moderate Risk, 3 = Great Risk
